# Supplementary material for: Comparative multiomics analysis of cell physiological state after culture in a basket bioreactor
Source: Sci Rep. 2022 Nov 23;12:20161. doi: 10.1038/s41598-022-24687-4 (PMC9686226; doi:10.1038/s41598-022-24687-4)

6:81210748:-@NW_023666042.1:81210036:81210109:-@NW_023666042.1:81195893:81195962:-@NW_0236660[illegible]

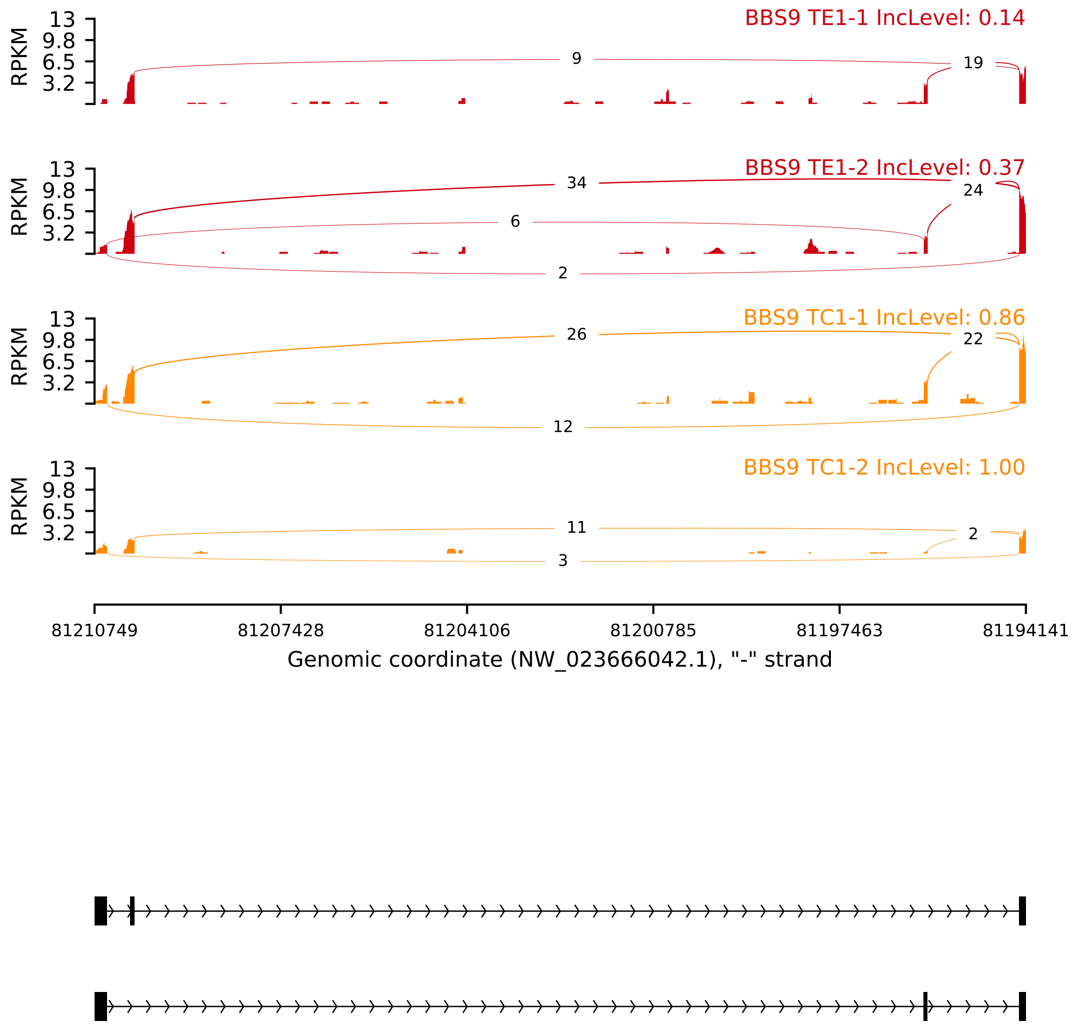

Supplement: Supplementary file 1 — Supplementary Information 1. [file 41598_2022_24687_MOESM1_ESM.zip › raw data/Transcriptomic raw data/7.AS/2.dasplot/TE1vsTC1/MXE/12_BBS9_NW_023666042.1_81210526_81210748_-@NW_023666042.1_81210036_81210109_-@NW_023666042.1_81195893_81195962_-@NW_023666042.1_81194139_81194261_-.pdf]
